# Association of IL-6 174G/C (rs1800795) and 572C/G (rs1800796) polymorphisms with risk of osteoporosis: a meta-analysis

**DOI:** 10.1186/s12891-020-03334-x

**Published:** 2020-05-28

**Authors:** Bin Chen, Hong-zhuo Li

**Affiliations:** 1 Changzhi Medical College, No. 161, Jiefangdong Street, Changzhi, 046000 Shanxi Province China; 2 Department of Orthopaedics, Heping Hospital Affiliated to Changzhi Medical College, Yanannan Road, Changzhi, 046000 Shanxi China

**Keywords:** IL-6, Single nucleotide polymorphism, Osteoporosis, Meta-analysis

## Abstract

**Background:**

Several studies have been performed to investigate association between IL-6 174G/C (rs1800795) and 572C/G (rs1800796) gene polymorphisms and osteoporosis predisposition. However, the results were conflicting. So, we performed a meta-analysis designed to provide more reliable results for the association between IL-6 gene polymorphisms and osteoporosis.

**Methods:**

Studies were searched using PubMed, EMBASE, the Cochrane Library and Wanfang electronic databases. The odds ratios (ORs) and 95% confidence intervals (CIs) were calculated to evaluate the association between IL-6 174G/C (rs1800795) and 572C/G (rs1800796) gene polymorphisms and osteoporosis risk. The false-positive report probabilities (FPRP) test and the venice criteria were used to assess the credibility of statistically significant associations.

**Results:**

A total of 9 studies with 1891 osteoporosis patients and 2027 healthy controls were included in current meta-analysis. Overall, The IL-6 174G/C (rs1800795) gene polymorphism was insignificantly associated with osteoporosis vulnerability. For IL-6 572C/G (rs1800796), statistically significant elevated osteoporosis vulnerability was found in IL-6 572C/G additive model (OR = 2.25, 95% CI: 1.55–3.26), dominant model (OR = 1.42, 95% CI: 0.78–2.56) and recessive model (OR = 1.96, 95% CI: 1.36–2.83). However, the IL-6 572C/G C allele was found to be associated with reduced susceptibility to osteoporosis (OR = 0.76, 95% CI: 0.56–1.04). When excluding studies that did not conform to HWE, the results did not change significantly. Further, when we evaluated the credibility of the positive results of the current meta-analysis, we identified less credible positive results in IL-6 572C/G recessive and additive model.

**Conclusion:**

In conclusion, IL-6 572C/G GG genotype may be associated with increased risk of osteoporosis.

## Background

Osteoporosis, one of the most common disorder, which is characterized by low bone mineral density (BMD) and degradation of bone microstructure, leading to an increased risk of fractures [1]. According to WHO standard, osteoporosis was defined as bone mineral density below 2.5 standard deviation of the average level of healthy adults. With the extension of human lifespan, more and more elderly people suffer from osteoporosis. Approximately 1.5 million new cases of osteoporotic fractures were reported every year worldwide, placing a huge financial burden on patient families [2]. It has become a public health problem in the world.

Several factors contribute to the pathogenesis of osteoporosis, such as exercise, age, sex, diet etc. [3]. In addition, genes and gene polymorphisms may also play an important role in osteoporosis predisposition [4]. Such as, In a study of familial diseases, bone mineral density was found to be highly heritables: 60–90% of BMD in the population is genetically determined [5]. Many genes were thought to be linked to osteoporosis and bone density. Those genes include estrogen receptor (ESR), calcitonin receptor (CTR), vitamin D receptor (VDR) and interleukin 6 (IL-6), etc. [6–8]. However, these risk genes can only explain part of the heritability of osteoporosis, and more variants have yet to be identified.

IL-6 gene is a multifunctional cytokine, located on human chromosome 7p21, which can stimulate the formation and absorption of bone cells [9–11]. Clinical studies had shown that the expression of IL-6 mRNA in bone explants of patients with osteoporotic vertebral fractures is enhanced [12]. Furthermore, some studies had found that IL-6 and other inflammatory cytokines can potentially up regulate the expression of RANKL on osteoblasts, accelerate the signal transduction of RANKL, and directly lead to bone destruction [13]. Recently, it has been discovered that functional polymorphisms of the IL-6 promoter,such as the C allele of the G-174C polymorphism, were associated with reduced promoter activity and plasma IL-6 levels, leading to reduced bone density [14–16]. Since Nordstrom first reported the link between IL-6 and susceptibility to osteoporosis [8], many related studies had also been published, but their results were conflicting. Such as, research by Ji Y F et al. revealed that G allele of rs1800796 was associated with increased risk of osteoporosis, while IL-6 174G/C was not significantly associated with osteoporosis [17]. However, the study of Magana et al. suggested that the increase of BMD is related to IL-6 174G/C, but not rs1800796 [18]. We consider that it may be related to the size of the sample size, the quality of the study, and whether the study conform to HWE. In view of this, we performed a meta-analysis in the hope of providing more reliable results for this association.

## Methods

### Search strategy

We performed the meta-analysis according to the guidelines of the PRISMA group [19]. Literature search was performed using PubMed, EMBASE, the Cochrane Library and Chinese Wanfang Data Knowledge Service Platform. The following search terms were applied in PubMed: (Interleukin-6 or IL-6) and (variant or variation or polymorphism) and (osteoporosis or osteoporoses). Language was not restricted in the current meta-analysis. The search deadline is March, 2020.

### Inclusion and exclusion criteria

Eligible publications were selected according to the following criteria: (1) case–control study; (2). case and control groups provid detailed genotype frequencies; (3) study must assess the association between IL-6 polymorphism and osteoporosis predisposition. Study excluded if it is a case report, duplicate or incomplete data, animal experiments, meta-analysis, and so on.

### Data extraction

According to the established inclusion and exclusion criteria, information was collected independently by two investigators. Potential differences were judged by a third system reviewers if necessary. The information collected was as follows: first author’s surname, year of publication, country, ethnicity, age, menopausal status, matching, diagnostic criteria of osteoporosis, sample size, and genotype frequencies.

### Quality assessment

The quality of individual studies was assessed independently by two researchers. We designed a study quality assessment criteria by referring to two previous meta-analyses [20, 21]. The total score of the scoring standard is 20 points. They were considered as high quality studies if quality scores were ≥ 12, while scores of ≤10 were regarded as low quality. The score between them is regarded as medium quality. Detailed scoring criteria were listed in Table 1.

### Statistical analysis

The crude odds ratios (ORs) and their 95% confidence intervals (CIs) were applied to evaluate the association between IL-6 polymorphisms on osteoporosis risk. Genotypes were assessed by the following models: allele model, additive model, dominant model and recessive model. Heterogeneity between studies assessed by *Q* test and *I*^*2*^ metric. Statistically significant heterogeneity between studies was considered, if *P* < 0.10 and *I*^*2*^ > 50% [22]. Meantime, the random- effects model were selected to pool results [23], if not, a fixed-effects model was used [24]. The source of heterogeneity was estimated by meta-regression analysis. Sensitivity analysis was performed according to two methods: first, a single study was removed each time, second, studies that do not conform to HWE and unmatched were removed [25]. Publication bias was determined based on Begg’s funnel plot [26] and Egger’s test (significant publication bias was considered if *P* < 0.05) [27]. Furthermore, the false-positive report probabilities (FPRP) test [28] and the Venice criteria [29] was used to evaluate the credibility of statistically significant associations. All statistical analyses were conducted using Stata 12.0 software.

## Results

### Description of included studies

**Fig. 1:**
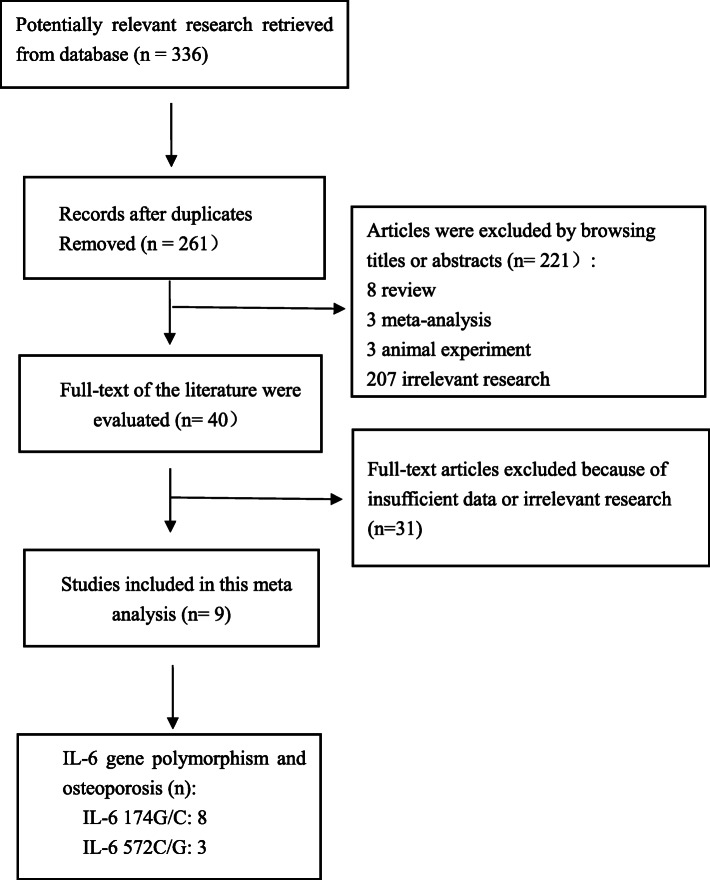
Flow diagram of the literature search

According to the pre-designed search strategy, 336 potential related studies were retrieved. Among them, 297 articles were excluded by reading titles or abstracts. Further, 30 studies that were irrelevant or did not provide genotypic data were excluded by detailed evaluation. Finally, 9 studies met inclusion and exclusion criteria (involving 1891 osteoporosis patients and 2027 healthy controls) [8, 17, 18, 30–35], of which 8 studies explored the relationship between IL-6 174G/C (rs1800795) and osteoporosis, 3 studies reported IL-6 572C/G (rs1800796) and osteoporosis predisposition. In addition, according to the quality evaluation criteria of our design, five studies were found to be of high quality, while the other four were of medium quality (as shown in Table 2). Furthermore, in all the included studies, two studies did not conform to HWE [8, 31]. The genotype frequencies of IL-6 174G/C, 572C/G and HWE test results were shown in Tables 3, 4. The selection process of the study is shown in Fig. 1.

**Table 1:** Scale for quality assessment of molecular association studies

Criterion	Score
Source of case	
Selected from population	2
Selected from hospital	1
Not described	0
Source of control	
Population-based	3
Blood donors or volunteers	2
Hospital-based	1
Not described	0
Ascertainment of osteoporosis	
**WHO**	**2**
Diagnosis of osteoporosis by patient medical record	1
Not described	0
Ascertainment of control	
Controls were tested to screen out	2
Controls were subjects who did not report osteoporosis, no objective testing	1
Not described	0
Matching	
Controls matched with cases by age and sex	2
Controls matched with cases only by age or sex	1
Not matched or not described	0
Genotyping examination	
Genotyping done blindly and quality control	2
Only genotyping done blindly or quality control	1
Unblinded and without quality control	0
Specimens used for determining genotypes	
Blood cells or normal tissues	1
Tumor tissues or exfoliated cells of tissue	0
HWE	
HWE in the control group	1
Hardy-Weinberg disequilibrium in the control group	0
Association assessment	
Assess association between genotypes and osteoporosis with appropriate statistics and adjustment for confounders	2
Assess association between genotypes and osteoporosis with appropriate statistics without adjustment for confounders	1
Inappropriate statistics used	0
Total sample size	
> 500	3
200–500	2
< 200	1
HWE: Hardy-Weinberg equilibrium

**Table 2:** General characteristics and quality scores of studies included in current meta-analysis

First author/Year	Country	Race	Gender	Cases						Controls					score
				N	Age (Mean ± SD) yrs (min-max)yrs	Menopause	BMD site	Diagnosis M	Matching	N	Healthy	Age (Mean ± SD) yrs (min-max)yrs	Menopause	BMD site	
Ji Y F, 2019	China	East Asia	Female	758	65.5 ± 16.1	PSM	LS-fn	Ne	age, sex	766	Yes	66.7 ± 17.0	PSM	LS-fn	14
Eftekhari H, 2018	Iran	West Asia	Female/Male	181	68 ± 7.21	PSM	LS-fn	WHO	age, sex	116	Yes	64 ± 5.44	PSM	LS-fn	17
Deveci D, 2012	Turkish	Caucasian	Female	201	57 ± 7	PSM	LS-fn	WHO	age, sex	155	Yes	57 ± 6	PSM	LS-fn	14
Czerny B, 2010	Poland	Caucasian	Female	226	63.3 ± 5.1	PSM	LS-fn	WHO	age, sex	224	Yes	64.8 ± 6.3	PSM	LS-fn	14
Breuil V, 2009	French	Caucasian	Female	92	70 ± 7.4	PSM	LS-fn	WHO	age, sex	69	Yes	64.1 ± 7.7	PSM	LS-fn	11
Magaña JJ, 2008	Mexican	Caucasian	Female	70	34.3 ± 10.2	Pre	LS	BMD values	age, sex	70	Yes	34.3 ± 10.2	Pre	LS	11
Dincel E, 2008	Turkish	Caucasian	Female/Male	21	74.47 ± 8.91	Ne	Fn	BMD values	age	21	Yes	75.47 ± 7.44	Ne	Fn	10
Kusek J, 2008	Poland	Caucasian	Female	110	58.5 ± 5.9	PSM	LS	WHO	sex	62	Yes	58.5 ± 5.9	PSM	LS	11
Nordstrom A, 2004	Sweden	Caucasian	Female	232	75 ± 0	PSM	LS-fn	BMD values	age, sex	544	Yes	75 ± 0	PSM	LS-fn	16

*Ne* not available, *PSM* Postmenopausal, *Pre* Premenopause, *LS* Lumbar spine, *Fn* Femoral neck

**Table 3:** Characteristics of the studies examinating the effects of IL-6 174G/C genes on osteoporosis risk

First author/Year	Ethnicity	Menopause	IL-6 174G/C genotype distribution						HWE	
			Case			Control			Chi-square test	*P*
			GG	GC	CC	GG	GC	CC		
Ji Y F, 2019	East Asia	PSM	399	285	74	440	270	56	2.613	0.106
Deveci D, 2012	Caucasian	PSM	127	50	24	93	31	31	42.528	0
Czerny B, 2010	Caucasian	PSM	67	126	33	76	103	45	0.872	0.3503
Breuil V, 2009	Caucasian	PSM	34	47	11	30	30	9	0.12	0.7293
Magaña JJ, 2008	Caucasian	Pre	56	14	0	42	25	3	0.09	0.7645
Dincel E, 2008	Caucasian	Ne	0	10	10	0	7	10	1.143	0.2851
Kusek J, 2008	Caucasian	PSM	24	63	23	12	36	14	1.637	0.2007
Nordstrom A, 2004	Caucasian	PSM	68	121	43	167	246	131	4.565	0.0326

### Quantitative synthesis

**Fig. 2:**
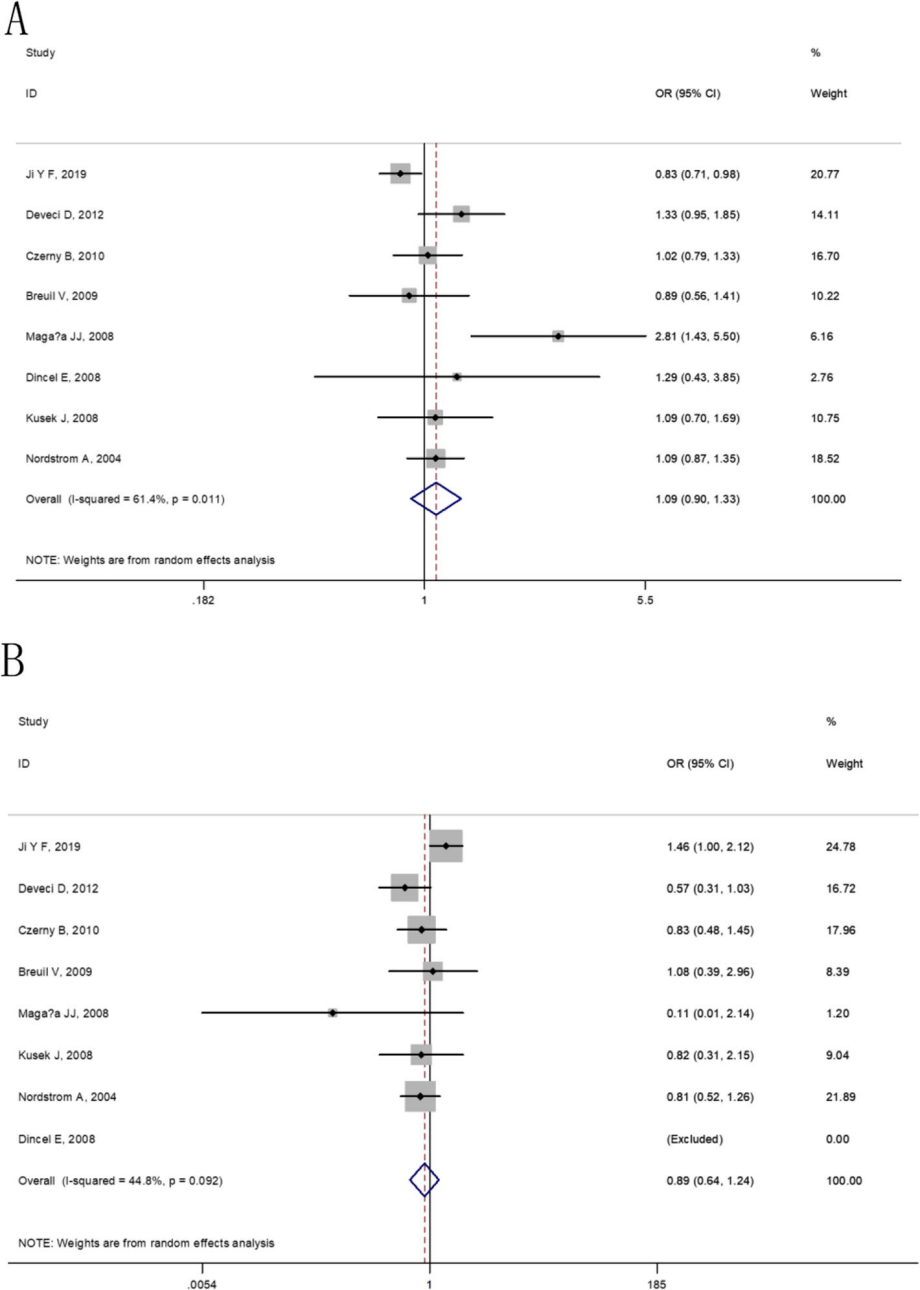
The forest plots on the association between IL-6 174G/C polymorphism and osteoporosis risk in overall (**a**: allele model, **b**: additive model)

**Fig. 3:**
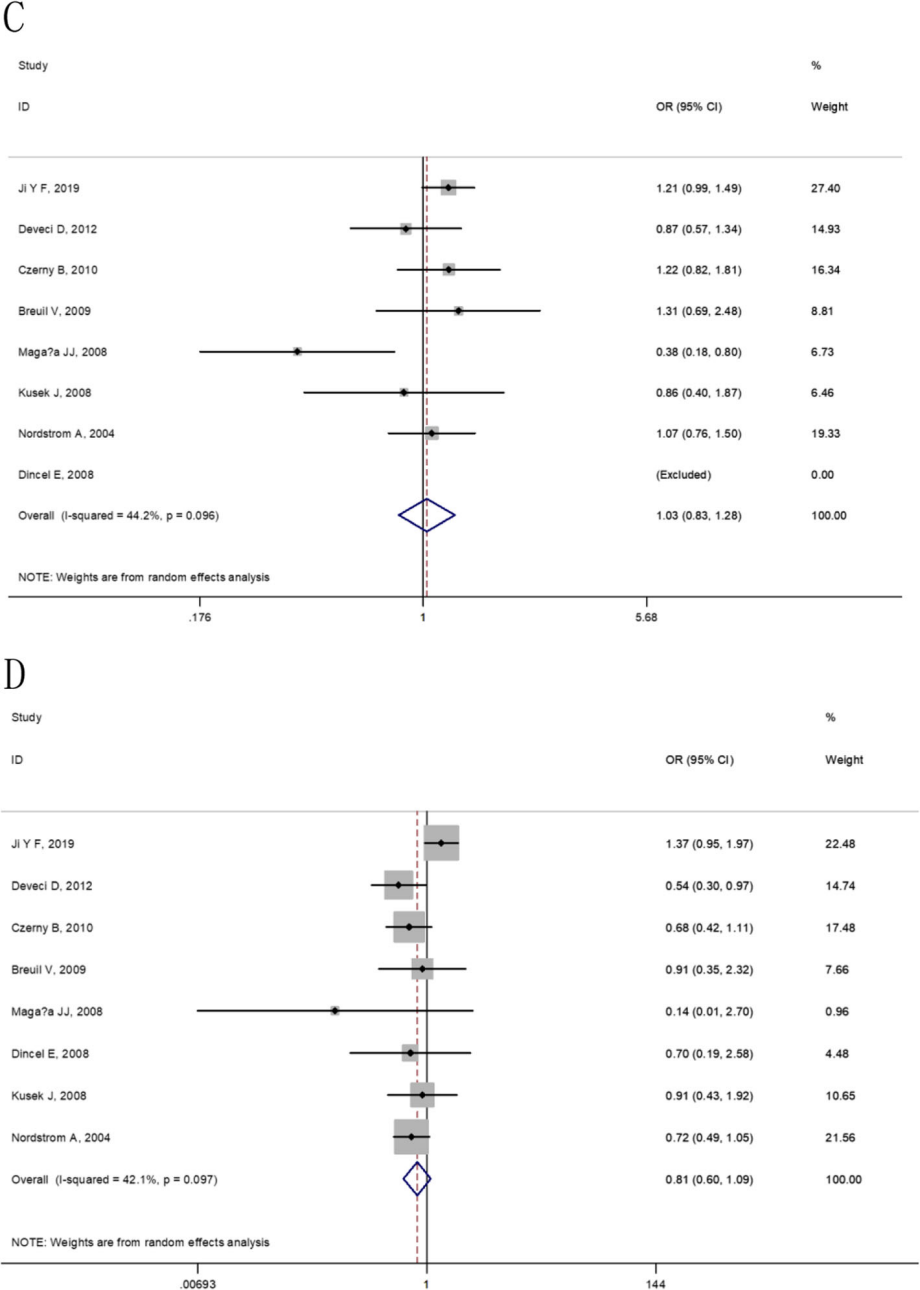
The forest plots on the association between IL-6 174G/C polymorphism and osteoporosis risk in overall (**c**: dominant model, **d**: recessive model)

**Fig. 4:**
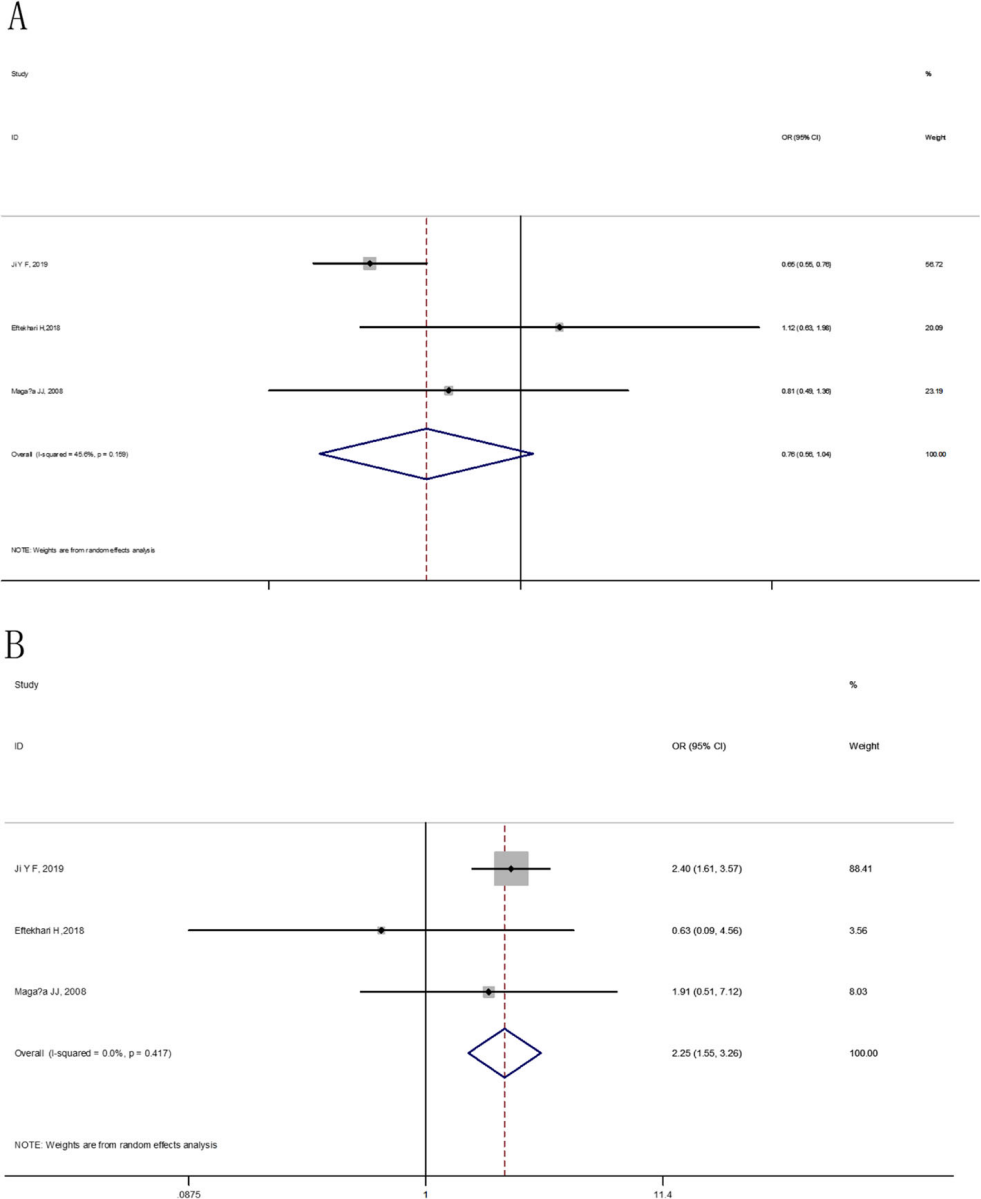
The forest plots on the association between IL-6 572C/G polymorphism and osteoporosis risk in overall (**a**: allele model, **b**: additive model)

**Fig. 5:**
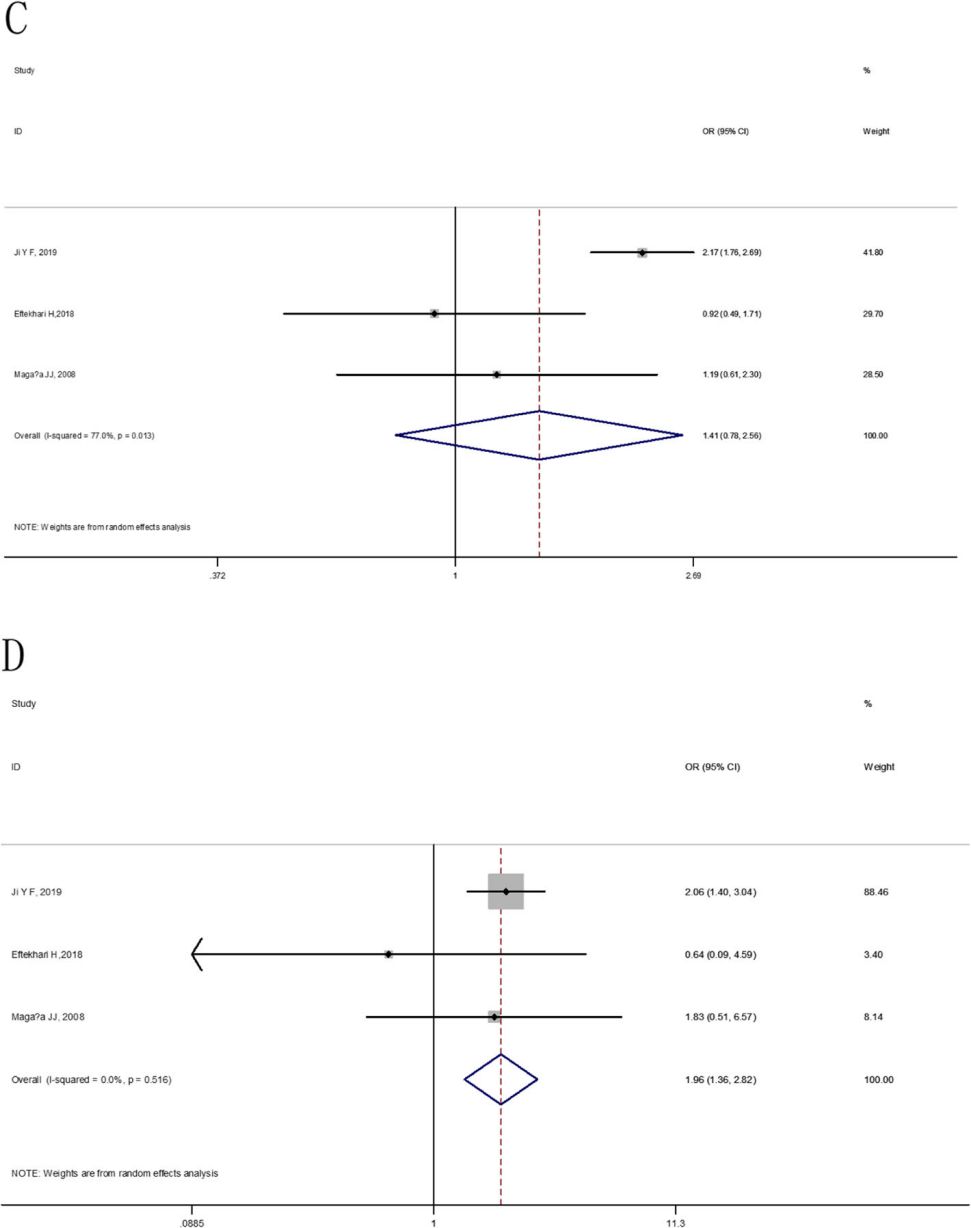
The forest plots on the association between IL-6 572C/G polymorphism and osteoporosis risk in overall (**c**: dominant model, **d**: recessive model)

At the overall analysis, The IL-6 174G/C (rs1800795) gene polymorphism was insignificantly associated with osteoporosis vulnerability in four genetic model comparisons (allele model: OR = 1.09, 95% CI: 0.90–1.33, additive model: OR = 0.89, 95% CI: 0.64–1.24, dominant model: OR = 1.03, 95% CI: 0.83–1.28 and recessive model: OR = 0.81, 95% CI: 0.60–1.09), (as shown in Table 5 and Figs. 2, 3). For IL-6 572C/G (rs1800796), statistically significant elevated osteoporosis vulnerability was found in IL-6 572C/G additive model (OR = 2.25, 95% CI: 1.55–3.26), dominant model (OR = 1.42, 95% CI: 0.78–2.56) and recessive model (OR = 1.96, 95% CI: 1.36–2.83). However, the IL-6 572C/G C allele was found to be associated with reduced susceptibility to osteoporosis (OR = 0.76, 95% CI: 0.56–1.04), (as shown in Table 5 and Figs. 4, 5).

**Table 4:** Characteristics of the studies evaluating the effects of IL-6 572 C/G genes on osteoporosis risk

First author/Year	Ethnicity	Menopause	IL-6 572 C/G (rs1800796) genotype distribution						HWE	
			Case			Control			Chi-square test	*P*
			CC	CG	GG	CC	CG	GG		
Ji Y F, 2019	East Asia	PSM	377	300	81	469	255	42	0.888	0.3461
Eftekhari H,2018	West Asia	Ne	152	27	2	96	18	2	1.071	0.3006
Magaña JJ, 2008	Caucasian	Pre	33	30	7	36	30	4	0.489	0.4843

### Heterogeneity and sensitivity analyses

The obvious heterogeneity between studies was observed in the current meta-analysis. Then, we evaluated the sources of heterogeneity by means of meta regression analysis. In IL-6 174G/C, the results suggested that menopause was the source of heterogeneity (*p* = 0.034 for Menopause vs Non-menopausal). Regretfully, we did not find the source of IL-6 572C/G dominant model heterogeneity.

Sensitivity analysis was estimated by applying two methods. First, the pool ORs did not change significantly when removing one study at a time to evaluate the robustness of the current meta-analysis. Furthermore, the results have not changed significantly when we restricted HWE and matching studies (as shown in Table 6) .

**Table 5:** The results of the meta-analysis for the association between IL-6 gene polymorphisms and osteoporosis

Genetic Model	N	Test of association		Tests for heterogeneity		Egger’s test
		OR (95%CI)	*p*_*h*_	*P value*	*I*^*2*^	*P*
IL-6 174G/C8						
G VS C		1.09 (0.90–1.33)	0.369	0.011	61.40%	0.076
CC VS GG		0.89 (0.64–1.24)	0.48	0.092	44.80%	0.171
GC + CC VS GG		1.03 (0.83–1.28)	0.812	0.096	44.20%	0.125
CC VS GG + GC		0.81 (0.60–1.09)	0.162	0.097	42.10%	0.272
IL-6 572C/G3						
C VS G		0.76 (0.56–1.04)	0.083	0.159	45.60%	0.226
GG VS CC		2.25 (1.55–3.26)	0	0.417	0.00%	0.306
CG + GG VS CC		1.42 (0.78–2.56)	0.253	0.013	77.00%	0.153
GG VS CC + CG		1.96 (1.36–2.83)	0	0.516	0.00%	0.365

IL-6 174G/C: allele model: G VS C; additive model: CC VS GG; dominant model: GC + CC VS GG; recessive model: CC VS GG + GC

IL-6 572C/G: allele model: C VS G; additive model: GG VS CC; dominant model: CG + GG VS CC; recessive model: GG VS CC + CG

### Publication bias diagnosis

**Fig. 6:**
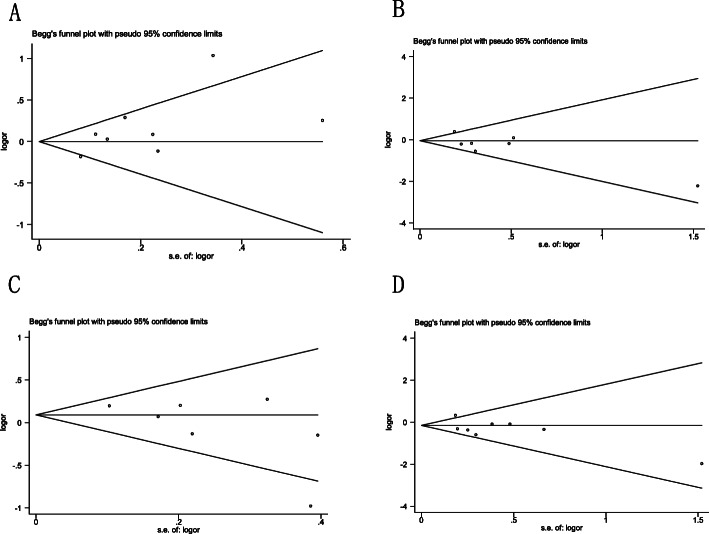
Begg’s funnel plot to assess publication bias on IL-6 174G/C polymorphism in overall population (**a**: allele model, **b**: additive model, **c**: dominant model, **d**: recessive model)

The publication bias was confirmed by Begg’s funnel plot and Egger’s test. No significant funnel asymmetry was found in all genetic models (Fig. 6). Similarly, There was no statistical evidence of publication bias based on Egger ‘s test results (*P* > 0.05 in all genetic models, as shown in Table 5).

### Credibility of the identified genetic associations

Genetic associations were classified as “positive results” when they met the following criteria [36]: (1) *P* value < 0.05 in at least two of the genetic models; (2) FPRP < 0.2; (3) statistical power > 0.8; (4) *I*^*2*^ < 50%. Associations were considered to be “less-credible positive results” if they met the following criteria: (1) *p* value < 0.05 in at least one of the genetic models; (2) FPRP > 0.2 or their statistical power was between 50 and 79% or *I*^*2*^ > 50%. Associations with *P* value > 0.05 were classified as “null” or “negative”. After credibility assessment, we identified “less-credible positive results” for IL-6 572C/G recessive and additive model in Overall. The credibility assessment results for the current meta-analyses were listed in Table 7.

**Table 6:** Pooled estimates of association of IL-6 174G/C and osteoporosis risk, only studies with matching, and studies conforming to HWE

Genetic Model	Test of association		Tests for heterogeneity	
	OR (95%CI)	*P*_*h*_	*P value*	*I*^*2*^
IL-6 174G/C				
G VS C	1.07 (0.82–1.39)	0.618	0.018	63.30%
CC VS GG	1.05 (0.71–1.57)	0.805	0.217	30.60%
GC + CC VS GG	1.01 (0.73–1.41)	0.942	0.05	57.80%
CC VS GG + GC	0.93 (0.65–1.34)	0.692	0.194	32.20%

**Table 7:** False-positive report probability values for the current meta-analysis

Variables	OR (95% CI)	*I*^*2*^*(%)*	*P*_*h*_	Statistical power		Prior probability of 0.01	
				0R = 1.2	OR = 1.5	0R = 1.2	OR = 1.5
IL-6 572C/G							
C VS G	0.76 (0.56–1.04)	45.60%	0.083	0.282	0.794	0.968	0.915
GG VS CC	2.25 (1.55–3.26)	0.00%	0	0	0.016	0.801	0.101
CG + GG VS CC	1.42 (0.78–2.56)	77.00%	0.253	0.288	0.572	0.988	0.977
GG VS CC + CG	1.96 (1.36–2.83)	0.00%	0	0.004	0.077	0.881	0.298

## Discussion

With the increasing aging of society, osteoporosis is becoming a serious social health problem, and have caused severe physical, psychological and economic burden to patients. Several factors contribute to the pathogenesis of osteoporosis. Among them, genetic factors play an important role in the histogenesis and development of osteoporosis [4]. Identification of potential pathogenic genes and their polymorphisms enables us to predict disease risk and take corresponding preventive measures. Interleukin-6 is one of the candidate genes due to it can potentially up regulate the expression of RANKL on osteoblasts, accelerate the signal transduction of RANKL, and directly lead to bone destruction [13]. There are some studies trying to figure out the association between IL-6 gene polymorphism and osteoporosis risk, while these results acquired were conflicting. The limited sample size of a single study is considered as one of the reasons. To overcome this shortcoming, meta-analysis is a competent alternative [37].

A total of 9 studies involving 1891 osteoporosis patients and 2027 healthy controls were included in the current meta-analysis, of which 8 studies evaluated the association between IL-6 174G/C polymorphism and osteoporosis, 3 studies related to IL-6 572C/G (rs1800796). Overall, We observed the higher osteoporosis risk in IL-6 572C/G additive, dominant and recessive model. However, the IL-6 572C/G C allele was associated with reduced osteoporosis risk. For IL-6 174G/C, the pooled odds rations indicate it was insignificantly associated with risk of developing osteoporosis in four genetic model comparisons. Furthermore, the current meta-analysis were performed by applying different genetic models, at the cost of multiple comparisons, in which case, the pooled *P*-value must be adjusted [37]. In the Venice criteria, statistical power and *I*^2^ were important indicator [38]. Hence, the false-positive report probabilities (FPRP) test [28] and the Venice criteria [29] were used to evaluate the credibility of statistically significant associations. Finally, we identified “less credible positive results” in IL-6 572C/G recessive and additive model. Heterogeneity was also observed in IL-6 174G/C allele model and IL-6 572C/G dominant model. We explore the source of heterogeneity using meta regression analysis. The results suggested that menopause was the source of heterogeneity. In addition, in molecular epidemiological studies, small sample studies are more likely to generate random errors and biases, and are more likely to produce false positive results [29]. Positive research results are more likely to be published, which can lead to publication bias. As shown in Fig. 6 in the current meta-analysis, the slight asymmetry of the funnel plot is caused by the study of small samples. However, due to the limited number of studies on IL-6 572C/G and osteoporosis risk, Begg’s funnel plot was not performed to explored publication bias.

It is worth noting that two previous meta-analyses of IL-6 174G/C and steoporosis risk have been published [39, 40]. Ni Y et al’s meta-analysis included 4 articles including 800 case groups and 900 control groups, and the results showed that the IL-6 CC genotype was significantly associated with a reduced risk for osteoporosis [39]. The examination of 12 studies by Fajar et al. indicated that 174G/C C allele and CC genotype may significant decreased osteoporosis risk [40]. However, when we carefully examined these two meta-analyses, we found that 6 articles [41–46] were incorrectly included in the study of Fajar et al. Such as Garnero et al. explored the relationship between IL-6 and BMD in premenopausal and postmenopausal healthy people. There was no osteoporosis in the case group [41]. Similarly, the case group in the Lee j et al. study was adolescents with idiopathic scoliosis [45], and Korvala et al. studied stress fractures in military personnel, not osteoporotic or osteoporotic fracture [44]. The other three studies did not provide detailed case and control genotype data [42–44]. So the results are not credible. In the meta-analysis of Ni Y et al., we found that the quality of the literature was not evaluated, no statistical power was calculated, and *p*-value was not adjusted after multiple comparisons. In order to overcome these shortcomings, the current meta-analysis was performed.

The advantages of current meta-analysis as follows: (1) this is the first meta-analysis to investigate the association between IL-6 572C/G polymorphisms and osteoporosis predisposition; (2) we performed a quality assessment of the literature to ensure the credibility of the pool results; (3) *p*-value was adjusted after multiple comparisons; (4) we conducted sensitivity analysis to test the stability of the current meta-analysis; (5) compared with previous meta-analysis, the current meta-analysis has a larger sample size. However, the current meta-analysis still has some defects. First, we have only investigated the relationship between the individual gene polymorphism of IL-6 and osteoporosis. In order to fully elucidate the pathogenesis of osteoporosis, it is necessary to study the combined role of these related genes. Second, the limited sample size of IL-6 572C/G may be the reason for the weak statistical power, so a larger sample size is needed to verify our results. Finally, due to the limited number of studies, we did not performed subgroup analysis.

## Conclusion

In conclusion, IL-6 572C/G GG genotype may be associated with increased risk of osteoporosis. The question of whether and how rs1800795 affect osteoporosis in postmenopausal women requires further investigation.

## Data Availability

The datasets supporting the conclusions of this article are included within the article and additional tables.

## Abbreviations

HWE: Hardy-Weinberg equilibrium
IL-6: Interleukin-6
OR: Odds ratio
95% CI: 95% confidence interval
PRISMA: Preferred Reporting Items for Systematic Review and Meta-Analyses
FPRP: False-positive report probabilities
BMD: Bone mineral density
PSM: Postmenopausal
Pre: Premenopause
LS: Lumbar spine
Fn: Femoral neck
